# The DNA Damage Inducible SOS Response Is a Key Player in the Generation of Bacterial Persister Cells and Population Wide Tolerance

**DOI:** 10.3389/fmicb.2020.01785

**Published:** 2020-08-04

**Authors:** Zdravko Podlesek, Darja Žgur Bertok

**Affiliations:** Department of Biology, Biotechnical Faculty, University of Ljubljana, Ljubljana, Slovenia

**Keywords:** SOS response, persisters, tolerance, biofilms, bacterial pathogens/opportunists

## Abstract

Population-wide tolerance and persisters enable susceptible bacterial cells to endure hostile environments, including antimicrobial exposure. The SOS response can play a significant role in the generation of persister cells, population-wide tolerance, and shielding. The SOS pathway is an inducible DNA damage repair system that is also pivotal for bacterial adaptation, pathogenesis, and diversification. In addition to the two key SOS regulators, LexA and RecA, some other stressors and stress responses can control SOS factors. Bacteria are exposed to DNA-damaging agents and other environmental and intracellular factors, including cigarette smoke, that trigger the SOS response at a number of sites within the host. The *Escherichia coli* TisB/IstR module is as yet the only known SOS-regulated toxin–antitoxin module involved in persister formation. Nevertheless, the SOS response plays a key role in the formation of biofilms that are highly recalcitrant to antimicrobials and can be abundant in persisters. Furthermore, the dynamic biofilm environment generates DNA-damaging factors that trigger the SOS response within the biofilm, fueling bacterial adaptation and diversification. This review highlights the SOS response in relation to antimicrobial recalcitrance to antimicrobials in four clinically significant species, *Escherichia coli*, *Pseudomonas aeruginosa*, *Staphylococcus aureus*, and *Mycobacterium tuberculosis*.

## Introduction

Bacteria are constantly exposed to a changing and stressful environment. The coordinated responses of global regulatory systems enable bacteria to survive and adapt to numerous environmental stresses (Foster, 2007).

Antibiotic resistance is one of the greatest threats to global health. Resistance allows growth in the presence of antibiotics due to genetic alterations that increase the minimal inhibitory concentration (MIC). Nevertheless, other mechanisms such as (i) population-wide tolerance, (ii) persisters, subpopulations characterized by a transient non-growing state and transient tolerance to lethal concentrations of antimicrobials (Wilmaerts et al., 2019), and (iii) shielding enable their survival in the presence of antimicrobial agents. Persisters play a pivotal role in chronic bacterial infections and the evolution of antibiotic resistance (Goormaghtigh and Van Melderen, 2019). Multifactorial and redundant molecular mechanisms are involved in the generation and the survival of persisters and tolerant cells (Molina-Quiroz et al., 2018; Trastoy et al., 2018). The most significant are global stress response, SOS response, oxidant tolerance, toxin–antitoxin system (TA), quorum sensing, energy metabolism, and drug efflux pumps (Dörr et al., 2009; Kwan et al., 2013; Harms et al., 2016; Trastoy et al., 2018). Recent studies have revealed that the SOS response plays significant roles beyond DNA damage repair. The goal of this review is to provide an overview of the SOS response in relation to persisters, tolerance, and shielding, highlighting several significant human pathogens.

## The SOS Response

The SOS response is an inducible DNA repair pathway controlled by two key regulators, LexA, a repressor, and RecA, an inducer. Upon DNA damage, RecA is activated (RecA^∗^) by binding to single-stranded DNA (ssDNA), forming a nucleoprotein filament that stimulates self-cleavage of LexA leading to, in *Escherichia coli*, de-repression of more than 50 SOS genes. The response is induced by an increase in intracellular ssDNA generated when DNA polymerase stalls at a lesion while helicase continues unwinding DNA. The SOS response is temporally controlled; thus, firstly induced are high-fidelity repair mechanisms, followed by low-fidelity, damage tolerance pathways involving error-prone translesion DNA polymerases PolII (*polB*), PolIV (*dinB*), and PolV (*umuC*, *umuD*) that are active only after an extensive, persistent DNA damage. These polymerases enable the repair of irrepairable DNA lesions that block DNA replication (Butala et al., 2009; Maslowska et al., 2019).

Conjugative plasmid DNA transfer and transformation also induce the SOS response (Baharoglu et al., 2010; Baharoglu et al., 2012) due to the transfer/uptake of ssDNA.

Exogenous and endogenous triggers induce the SOS response. The exogenous triggers include UV irradiation, drugs, oxidants, and chemical mutagens. The induction of SOS response and mutagenesis by subinhibitory concentrations of antimicrobials has been demonstrated in several bacterial species: *Escherichia coli*, *Vibrio cholerae*, *Pseudomonas aeruginosa*, *Staphylococcus aureus*, and *Streptococci* species (Baharoglu and Mazel, 2011; Andersson and Hughes, 2014). The endogenous triggers are metabolic intermediates, stalled replication forks, and defects following recombination or chromosome segregation (Pennington and Rosenberg, 2007; McGlynn et al., 2012).

While the SOS response was initially recognized as regulating DNA damage repair, it plays a much broader role. The SOS error-prone polymerases promote an elevated mutation rate, generating genetic diversity and adaptation, including antibiotic resistance. Furthermore, the SOS response controlling the TA systems is significant for biofilms that exhibit increased antimicrobial recalcitrance, horizontal gene transfer (Guerin et al., 2009), intraspecies competition, and phenotypic variation (Maslowska et al., 2019).

## Other Bacterial Stress Responses Control SOS Factors

While RecA and LexA are the principal SOS regulators, other stress response pathways such as the general stress responses regulated by alternative sigma factors RpoS and RpoH, the stringent response, reactive oxygen species (ROS), and cAMP are also involved in the control of SOS factors/induction.

RpoS is a regulator of the general stress response as various stresses that inhibit growth provoke RpoS activity (Hengge, 2009). Furthermore, in *E. coli*, RpoS regulates Pol IV activity (Layton and Foster, 2003) and is a positive regulator of *polB* (Pol II) (Dapa et al., 2017).

While RpoH (σ^32^) controls the heat-shock response during exponential growth, its synthesis and activity is also induced by other conditions, leading to unfolded proteins (Morita et al., 2000). In *E. coli*, RpoH indirectly affects the levels of Pol V and Pol IV *via* the molecular chaperone GroE. The latter is governed by RpoH and interacts with and protects UmuC (DNA polymerase V) from degradation (Donnelly and Walker, 1992). GroE is also required for the normal and the induced levels of Pol IV, albeit indirectly (Layton and Foster, 2003).

The ROS are produced in metabolic pathways as well as by the immune response upon infection. Environmental agents such as UV, drugs including antimicrobial agents, and heat can provoke and increase the ROS levels that subsequently damage DNA, proteins, and lipids, inducing the SOS response. When more ROS are produced than eliminated, oxidative stress ensues (Zhao and Drlica, 2014).

The stringent response is a conserved global stress response to nutrient and iron starvation that involves the production of (p)ppGpp. Starvation activates the stringent response *via* induction of the *relA* and *spoT* genes to synthesize (p)ppGpp that subsequently affects transcription, translation, and replication. In *E. coli*, increased levels of (p)ppGpp alters transcription in response to nutrient starvation and other stresses, resulting in reduced growth rate (Ronneau and Hallez, 2019). (p)ppGpp can increase the pause time of transcription elongation complexes and arrest DNA replication forks by targeting DNA primase, exposing ssDNA and inducing the SOS response (Srivatsan and Wang, 2008). In *E. coli*, the stringent response indirectly induces the expression of *recA*, *ruvA*, *sbmC*, *sulA*, *umuD*, and *yebG* genes, which belong to the SOS regulon (Durfee et al., 2008).

The cyclic AMP receptor protein (CRP) is (primarily) a global transcription regulator of genes involved in carbon catabolite repression (Molina-Quiroz et al., 2018). Nevertheless, cAMP–CRP also regulates the genes involved in virulence, biofilm formation, and SOS response and negatively regulates persistence in uropathogenic *E. coli* (UPEC) cultures exposed to ampicillin (Molina-Quiroz et al., 2018).

## The SOS Response and Biofilms

Biofilms are structured bacterial communities attached to inert or living surfaces that create a protective environment for bacterial cells (Hall-Stoodley et al., 2004). While biofilm formation is an integral part of the prokaryotic life cycle, biofilms also cause biofilm-related diseases that are difficult to eradicate, e.g., urinary tract infections (UTI), chronic infections in cystic fibrosis (CF) patients, colonization of medical devices, and periodontal diseases (Costerton et al., 1999; Arciola et al., 2018). Biofilm formation is a highly regulated process depending upon numerous environmental and genetic factors (López et al., 2010; van Gestel et al., 2015; Qin et al., 2020). Biofilms are induced by antimicrobial stress. In biofilms, bacteria are able to survive a high-dose antibiotic treatment (Costerton et al., 1999). The recalcitrance of biofilms to antimicrobials is multifactorial. The extracellular matrix forms a mechanical barrier that prevents antibiotic diffusion. Furthermore, low oxygen and nutrient concentrations within the mature biofilms create niches with low bacterial metabolic activity. Up to 1% of bacterial cells in biofilms may be persister cells which, due to dormancy, are not affected by antimicrobials (Hall and Mah, 2017). Furthermore, high cell density within biofilms enhances horizontal gene transfer and competition that, together with accumulation of metabolic products, microaerobic areas, and oxidative stress, incite DNA damage and provoke the SOS response. Furthermore, phenotypic variants, e.g., small colony variants (SCVs) from biofilms, are quasi-dormant, slow growing, and very tolerant to host defenses and antimicrobials. SCVs attach strongly to surfaces, display increased production of exopoysaccharides, and may autoaggregate (Soares et al., 2019). They are potentially responsible for difficult-to-treat persistent infections, wherein bacteria persist in the host for prolonged periods of time despite antimicrobial therapy. Thus, the recalcitrance of biofilms to antimicrobials can be due to tolerance, when dispersed biofilm cells exhibit antibiotic sensitivity and low MIC, as well as resistance, characterized by increased MICs and a resistant phenotype of dispersed biofilm bacteria. The SOS response plays a significant role in biofilm formation but, in turn, in the dynamic biofilm environment, SOS-inducing factors that promote mutagenesis and diversification are generated.

## SOS-Controlled Persisters and Tolerance in Selected Bacterial Pathogens

### Escherichia coli

Persisters and antimicrobial tolerance have been most extensively studied in *E. coli*, a commensal of the gut but also a leading cause of intestinal and extraintestinal infections. One of the best-studied and most clear-cut example of persister cell formation involving the SOS system is activation *via* the class I toxin–antitoxin TisB/IstR module (Table 1). TisB is a small membrane-acting peptide that decreases the proton motive force and ATP levels, shutting down cell metabolism and inducing dormancy (Dörr et al., 2010). The *tisB* gene is repressed by LexA, while the IstR-1 antitoxin is constitutively expressed. The regulation of *tisB* mRNA translation is complex, encompassing inhibition by the RNA IstR-1 antitoxin as well as an inhibitory structure in the 5′UTR of the *tisB* mRNA. Upon DNA damage and SOS induction, *tisB* transcription strongly increases and exceeds that of the antitoxin IstR-1 (Berghoff et al., 2016).

**TABLE 1:** SOS response induced characteristics/factors associated with persisters/tolerance.

Pathogen	SOS-regulated characteristics/factors	References
*E. coli*	Toxin TisB	Berghoff et al., 2016
	From persisters/tolerance to resistance	Cohen et al., 2013
		Levin-Reisman et al., 2017
		Windels et al., 2019
		Barrett et al., 2019
	Maintenance of murine gut colonization	Samuels et al., 2019
	Intracellular biofilms in urinary tract infections	Lewis et al., 2016
	Enterobacterial blooms	Stecher et al., 2013
	Bacteriocin production→intraspecies competition→SOS induction	Cascales et al., 2007
*P. aeruginosa*	Motility/biofilm formation	Rodríguez-Rojas et al., 2012
	Bacterial cell aggregation	Secor et al., 2018
	Bacteriocin production→cell lysis→cell components for biofilm matrix	Turnbull et al., 2016
	SCV	Hui et al., 2014
*S. aureus*	Increased persister levels	Tong et al., 2015
	Biofilm formation	Berditsch et al., 2019
	Enhanced fibronectin binding protein	McCourt et al., 2014
	SCV	Lacoma et al., 2019
	Intracellular persisters	Peyrusson et al., 2020
*M. tuberculosis*	Survival, antibiotic resistance	Boshoff et al., 2003
	*Gyrase modulates SOS response, persisters	Choudhary et al., 2019
	*CarD modulates DNA damage, starvation, oxidative stress	Stallings et al., 2009
	*Cas 1 modulates DNA damage repair and other stress responses	Wei et al., 2019

SCV, small colony variant; *factors associated with SOS response/DNA damage.

Nevertheless, in *E. coli*, the SOS response is not only involved in persister cell formation but, in persisters, accelerates antibiotic resistance (Cohen et al., 2013; Levin-Reisman et al., 2017; Windels et al., 2019). Thus, from fluoroquinolone (FQ) persisters, the SOS response promotes resistance to unrelated antibiotics following a single FQ exposure (Barrett et al., 2019).

Conditions conducive to SOS induction are encountered by *E. coli* at various host anatomical sites. The SOS response plays a vital role for maintaining colonization of the murine gut by commensal *E. coli* with competing commensal organisms, a possible source of genotoxic stress (Samuels et al., 2019).

Urinary tract infections are a worldwide health concern caused mainly by UPEC strains. To provoke UTI, UPEC undergo a complex intracellular cycle (Lewis et al., 2016), forming biofilms within bladder epithelial cells and evading antimicrobial agents. In response, the infected cells produce nitric oxide that attacks DNA, inducing the SOS response. UTI frequently lead to chronic infection, and a persister subpopulation could be responsible for generating relapsing infections (Blango and Mulvey, 2010).

Biofilms are also associated with *E. coli* intestinal infections (Sharma et al., 2016). DNA damage can be provoked by host factors, e.g., bile salts, and by competing microbes. Furthermore, intestinal inflammation triggered by infection or the gut immune system involving ROS provokes the SOS response and dysbiosis, suppressing anaerobes and inciting *Enterobacteriaceae* overgrowth with competition for nutrients (Stecher et al., 2013).

Colicins are *E. coli* LexA-repressed bacteriocins that govern intraspecies competition. Nuclease colicins can, in sensitive *E. coli*, activate the SOS response *via* DNA degradation (Cascales et al., 2007). Furthermore, lysis of the producer-releasing colicin, as well as lysis of the sensitive target cell, provides material for bacterial shielding or biofilm matrix. Furthermore, a recent study showed that ampicillin-induced bacterial cell lysis provides a matrix of cell debris that shields viable cells from antimicrobial activity (Podlesek et al., 2016).

### Pseudomonas aeruginosa

*P. aeruginosa* is a ubiquitous Gram-negative bacterium and a major human opportunist. It is a particular threat for immunocompromised patients with CF, burns, or chronic wounds (Salter, 2015). The *P. aeruginosa* SOS response regulon is comprised of only 15 genes, including *recA*, *lexA*, and others involved in DNA repair and induced mutation. Nevertheless, the *P. aeruginosa* SOS response is complex as, upon induction, the LexA and two other regulons regulated by two LexA-like Ser-Lys dyad repressors, PrtR and PAO906, are also upregulated (Matsui et al., 1993; Michel-Briand and Baysse, 2002; Cirz et al., 2006). In the stressed lung environment, strains evolve *via* genomic mutations and rearrangements, resulting in intrapopulation phenotypic variation (Darch et al., 2015). One of the most important factors for the survival of *P. aeruginosa* is the capacity to develop biofilms in various environments, including chronic lung infections in individuals with CF, rendering antibiotics inefficient (Høiby et al., 2010). These biofilms also harbor abundant persisters (1%).

In *P. aeruginosa*, environmental factors, such as ROS from the inflammatory response, as well as DNA-damaging antibiotics induce biofilm formation *via* the SOS response (Rodríguez-Rojas et al., 2012). The initial event in biofilm formation is repression of motility, and the results indicate that, in *P. aeruginosa*, repression is driven by the LexA protein (Chellappa et al., 2013). In *Salmonella* spp., the activation of the SOS response increases the RecA concentration that subsequently and indirectly impairs swarming motility (Irazoki et al., 2016), while in *Clostridium difficile* the inactivation of the *lexA* gene provoked a reduction in swarming motility (Walter et al., 2015).

Aggregation is, in *P. aeruginosa*, also achieved at chronic infection sites by the depletion mechanism that requires neither biofilm assembly functions nor bacterial viability (Secor et al., 2018). Depletion aggregation markedly and rapidly increases antibiotic tolerance *via* the SOS response.

Pyocins, chromosomally encoded and SOS-regulated bacteriocins produced by most *P. aeruginosa* strains, also play a significant role in biofilm formation (Michel-Briand and Baysse, 2002). A recent study revealed that R-type pyocins play an important role in competition among *P. aeruginosa* strains and could be pivotal for the dominance of epidemic strains in CF patients (Oluyombo et al., 2019). In addition, when pyocins are released *via* cell lysis, chromosomal DNA and the other released components function as a matrix for biofilm formation (Turnbull et al., 2016).

While persisters are abundant among bacterial isolates from the lungs of chronic CF patients (Mulcahy et al., 2010), SCVs are also commonly isolated from *P. aeruginosa* infections. Interestingly, the Pf4 bacteriophage plays an important role in *P. aeruginosa* biofilm formation, stress tolerance, and formation of morphotypic variants such as SCV (Rice et al., 2009). The accumulation of reactive oxygen and nitrogen species and the exposure to sublethal concentrations of antibiotics in the biofilm may result in DNA lesions in Pf4, leading to the formation of superinfection SI phage, which subsequently selects for morphotypic variants, such as SCVs (Hui et al., 2014). These SCVs are usually non-motile and recalcitrant to several different classes of antibiotics (Malone, 2015). *In vitro* tests have shown that exposure to sublethal concentrations of aminoglycosides selects for SCVs.

Furthermore, in *P. aeruginosa*, the stringent response enhances antimicrobial tolerance in biofilms by preventing the accumulation of pro-oxidants and upgrading the antioxidant activity, thus reducing oxidative stress (Nguyen et al., 2011; Khakimova et al., 2013).

### Staphylococcus aureus

*S. aureus* is a major human bacterial pathogen that colonizes the nasopharynx and the skin of over one-third of the human population. It can also cause acute as well as persistent infections. Well characterized are biofilm infections on implanted medical devices (Tong et al., 2015). Pretreatment with sublethal concentations of antibiotics substantially increased the levels of persister cells (Johnson and Levin, 2013). Furthermore, in planktonic bacteria, exposure to subinhibitory concentrations of antibiotics induced switching to a biofilm lifestyle (Berditsch et al., 2019). SOS induction has also been shown to enhance the expression of fibronectin-binding protein (Bisognano et al., 2004) that facilitates attachment to host cells and biofilm formation (McCourt et al., 2014). Adhesion is the first step in biofilm formation or host cell invasion.

In *S. aureus*, only 16 LexA regulated genes have been identified, with one error-prone polymerase, UmuC (Cirz et al., 2007). The SOS response plays a key role in the emergence of antibiotic resistance, as well as the expression and the dissemination of virulence factors encoded on pathogenicity islands, SaPI.

Furthermore, in *S. aureus*, antibiotic-provoked SOS induction increases the formation of SCV. The mutagenic DNA repair pathway with the AddAB complex (termed RexAB in *S. aureus*), RecA and PolV, provokes subpopulations of SCVs resistant to H_2_O_2_ due to enhanced catalase production (Painter et al., 2015).

Tobacco smoking represents the leading preventable cause of death worldwide. A recent study showed that, in *S. aureus*, cigarette smoke provoked growth inhibition, increased biofilm formation, increased the invasion of and the persistence within bronchial alveolar epithelial cells, as well as enhanced the mutation frequency, resulting in a significant increase in gentamicin-resistant SCV. SOS DNA mutagenic repair was shown to induce SCV formation (Lacoma et al., 2019).

Recently, the antibiotic treatment of *S. aureus* was found to elicit intracellular persisters characterized by profound transcriptomic reprogramming with active adaptive responses, including the SOS response (Peyrusson et al., 2020). Furthermore, exposure to a single antibiotic leads to tolerance to several antibiotic classes with unrelated targets. The intracellular survival of *S. aureus* is considered as a significant factor responsible for recurrent infections (Garzoni and Kelley, 2011).

### Mycobacterium tuberculosis

Tuberculosis is one of the leading causes of death worldwide, with a rapid emergence and spread of drug-resistant strains (Choudhary et al., 2019). During infection, the host employs an array of stresses to restrain *M. tuberculosis* (Mtb) proliferation. Nevertheless, Mtb may persist for decades, indicating the possession of efficient molecular mechanisms to resist host-inflicted damage.

In mycobacteria, two mechanisms control DNA damage repair, a relatively small LexA-regulated and a larger LexA-independent mechanism controlled by a ClpR factor (Smollett et al., 2012). The error prone α subunit of DNA-polymerase III encoded by *dnaE2* is required for persistence during infection and for the development of antibiotic resistance (Boshoff et al., 2003).

In contrast to *E. coli*, which harbors error-prone polymerases, II, IV, and V, Mtb, carries a split cassette, imuA′–imuB/dnaE2. ImuA′ and ImuB are Y-family DNA polymerases essential for induced mutagenesis and damage tolerance in conjunction with the C-family DNA polymerase DnaE2 (Warner et al., 2010). ImuB interacts with ImuA′ and the C-family DNA polymerase, DnaE2, as well as with the beta-clamp. Bacterial genomes are replicated by the DNA polymerase III α subunit (PolIIIα) that classifies into the C-family of DNA polymerases. Two major forms of PolIIIα are recognized, PolC and DnaE (three groups: DnaE1, DnaE2, DnaE3), inferred to have evolved by duplication (Koonin and Bork, 1996). An analysis of approximately 2,000 bacterial genomes showed that they harbor one or more homologs (Timinskas et al., 2014). DnaE2 is part of SOS-inducible mutagenic cassettes identified in many bacterial genomes, including *M. tuberculosis*. At least some DnaE3s are also error prone and SOS inducible (Boshoff et al., 2003).

Transcriptomic studies showed that various stress response regulons, including the SOS response and different TA genes, are positively regulated in Mtb persisters (Keren et al., 2011; Smollett et al., 2012).

Recently, the suppression of DNA gyrase was found to drastically affect intra- and extracellular Mtb growth. Interestingly, gyrase depletion in Mtb leads to the activation of RecA/LexA-mediated SOS response and drug tolerance *via* induction of a persister subpopulation (Choudhary et al., 2019).

Furthermore, (p)ppGpp (Prusa et al., 2018) and CarD (Stallings et al., 2009) play significant roles for the successful growth of *M. tuberculosis* in the host. Lack of CarD leads to killing of *M. tuberculosis* due to DNA damage, starvation, and oxidative stress (Stallings et al., 2009).

Biofilm growth is also significant for Mtb particularly during infection. Mtb spontaneously grows at the air–liquid interface, forming pellicle biofilms with elevated drug-tolerant persisters compared to planktonic cultures. The intrinsic drug tolerance of biofilm cells was found to determine the frequency of persisters and isonitrile lipopeptide necessary for the architectural development of Mtb biofilms (Richards et al., 2019).

The CRISPR-associated protein 1 (Cas1) is an endonuclease responsible for spacer integration into CRISPR arrays. Interestingly, Cas1 was found to be deleted in many specific drug-resistant strains. In recombinant *M. smegmatis*, Cas1 increased the sensitivity to multiple anti-tuberculosis drugs by reducing persistence during antimicrobial treatment. Cas1 also impaired the repair of DNA damage and altered the stress response of *M. smegmatis* (Wei et al., 2019).

## Concluding Remarks

Multiple redundant mechanisms are involved in the formation of persister cells, population-wide tolerance, and shielding. DNA damage and other stress pathways trigger the SOS response or control SOS factors in a number of host sites. As yet, only one SOS-regulated TA module has been shown to be involved in persister formation. Nevertheless, the SOS system is a key regulator of biofilm formation that protects bacterial communities against extreme environmental conditions, including antibiotic exposure. Furthermore, in biofilms, persisters and other phenotypic variants can be abundant. The conditions and the mechanisms within a biofilm generate factors that trigger the SOS response, fueling mutagenesis, horizontal gene transfer, competition, and diversification. The discussed four pathogens/opportunists provoke significantly persistent, hard-to-treat infections also due to persister cells and tolerance. Nevertheless, our current understanding of the means and the levels of the SOS response and its links with other global regulatory responses is still lacking. Therapies aimed at preventing persister formation but particularly at suppressing the SOS response, possibly targeting its inducer RecA, could greatly improve patient outcome.

## Author Contributions

DŽ provided the general concept. DŽ and ZP wrote the manuscript. Both authors contributed to the article and approved the submitted version.

## Conflict of Interest

The authors declare that the research was conducted in the absence of any commercial or financial relationships that could be construed as a potential conflict of interest.
